# Spasms and Convulsions

**Published:** 1895-08

**Authors:** A. W. Holcombe

**Affiliations:** Kokomo, Indiana


### SPASMS AND CONVULSIONS.

A. W. Holcombe, M. D., Kokomo, Indiana.

[concluded from page 341.]

Jaw, lower. Camph.
Limbs, clonic. Dolich.
Side, left. Glon., Ip., Calc-ph.
Side, right, left paralyzed. Art-vul.
Side, one. Cup.
Side, one, face and shoulder. *Aurant.*

*Attack caused or aggravated by*

Abortion, after. *Sec-c.*
Alcohol, abuse of. ALCOH., NUX-V.
Anger. NUX-V., Op. (*Cham*).
Anger, of nurse. Cham.
Blood, loss of. China.
Blows or concussions to head. Hyper.
Coitus, after. Cedron.
Climaxis, during. Lach.
Cold, from a. Indig.
Cold, from catching. *Nux-v.*
Contact, least. Bell., CIC-V.
Cooling off, while overheated. Art-vul.
Catarrh, suppresssed. Camph.
Dentition. ART-VUL., Coff-crd., Igt., Mag-ph., Millef., Pod., Stan., Verat-a., Zinc.
Dentition of eye-teeth. *Chlorum.*
Disturbed, when. Atr-sul.
Drink. Canth., Lyss., STRAM.
Eczema, suppressed. Kali-m.
Emotion, mental. Coff-crd., Igt., Kali-br., *Sul.*

Eruption, suppressed. Agar., Ant-t., CALC-C., CAMPH., CAUST., STRAM., SUL.
Eruption, before breaks out. CUP-AC.
Exanthema, at outset of. *Crot-hor.*
Fever and diarrhœa. ART-VUL., HYDR-AC.
Footsweat, checked. SIL.
Fright. AGAR., ART-VUL., Bell., BUFO, CALC-C., CAUST., CUP-AC., CUP., HYOS., IGT., OP., STRAM.
Fright, during menses. *Arg-nit.*
Grief. ARS., ART-VUL., HYOS., IGT.
Heart, valvular diseases of. CALC-ARS.
Indigestible food. IPEC.
Indigestion. NUX-V.
Injury, after. HYPER.
Injury to head. NAT-SUL.
Laughing or crying. Bell.
Labor, after. *Millef.*
Light, bright. CANTH., Lyss., STRAM.
Meals, after. HYOS., IGT., CALC-PH.
Measles, repercussion of. BRY.
Measles, after. CHAM.
Menses, suppressed from bathing. CALC-PH.
Menses, sudden checking of. COCC., GELS., *Millef.*
Menses, before. Carb-veg., IGT.
Menses, during. CEDRON., CIM., KALI-BR., LACH. (*Œnanthe*), PLAT., *Tarent.*
Menses, after. IGT.
Menses, at establishment of. *Caul.*
Menstrual disturbances. ART-VUL., Caul.
Motion. CUP-AC.
Moving limb. Ant-t.
Noise, sudden. *Sul.*
Onanism. BUFO, CALC-C., LACH.
Opening door. CIC-V.
Ovarian irritation. ATRO-SUL.
Pain. *Vespa*, Bell.
Parturition, during. CHIN-SUL.

Pregnancy. CIC-V.
Pressure on brain during delivery. HEPAR.
Pressure over solar plexus. NUX-V.
Puerperal state. HYOS., VERAT-V.
Puberty. CAUST.
Punishment, in children. IGT.
Religious excitement. VERAT-A.
Sexual indulgences, excessive. KALI-BR.
Scarlatina, suppression of. CAMPH.
Sleep, loss of. COCC.
Suppuration of internal parts. *Bufo.*
Swallow, any attempt to, speaking, current of air, sight or idea of fluids, running water, contact-light, noises or strong odors. LYSS.
Touch. *Sul.*
Trouble, domestic. STAPH.
Uterine irritation. TARENT., VIBURN-OP.
Vaccination, after. SIL.
Vomiting, during. GUAREA-TRICH.
Water, sound of running or falling. CANTH., LYSS.

*"Attacks prevented or ameliorated by"*

Lying on back. IGT.
Lying down. CUP., CUP-AC.
Riding in carriage. NIT-AC.
Water, putting feet in hot. BUFO.
Water, drinking cold. CAUST.

*Time of Attack.*

Two o'clock A. M. *Kali-br.*
Four o'clock A. M. KALI-BR.
Morning. ART-VUL.
Dawn. PLAT.
Day, during. CALC-ARS.
Evening. CALC-C., CAUST.
Noon, toward. Acon.

Night, at. ARG-NIT., ART-VUL., CALC-ARS., CALC-C., CAUST., CINA, MERC., *Sec-c.*, SIL.
Sleep, during. BUFO, CHLORAF., Cup., HYOS., IGT., LACH., *Op.*
Midnight, at. Cocc., BUFO.
Closing eyes, on, to sleep. HYDR-AC.
Table, at the. MAG-C.
Same hour, returns at. (CEDRON.), IGT.
Every seven days. *Chin-sul.*, Croc-sat.
New moon. BUFO, CAUST., Cup., KALI-BR., SIL.
Full moon. CALC-C., *Nat-m.*

